# Effect of Affective Temperaments Assessed by the TEMPS-A on the Relationship between Work-Related Stressors and Depressive Symptoms among Workers in Their Twenties to Forties in Japan

**DOI:** 10.1155/2012/469384

**Published:** 2012-09-06

**Authors:** Maki Tei-Tominaga, Tsuyoshi Akiyama, Yoshie Sakai

**Affiliations:** ^1^Department of Nursing, Hyogo University of Health Sciences, 1-3-6 Minatojima, Chuo-ku, Kobe 650-8530, Japan; ^2^Department of Neuropsychiatry, Kanto Medical Center, Nippon Telegraph and Telephone East Corporation, 5-9-22 Higashi-Gotanda, Shinagawa-ku, Tokyo 141-8625, Japan; ^3^Department of Literature, Atomi University, 1-9-6 Nakano, Niiza-shi, Saitama 352-8501, Japan

## Abstract

Relatively recently in Japan, *immature-type depression*, frequently classified in the bipolar II spectrum, has increased among workers in their twenties to forties. This study explored whether affective temperaments moderate the relationship between work-related stressors and depressive symptoms among this age group. In July 2004, self-administered questionnaires were distributed to all employees of a Japanese company. Eight hundred seventy-four employees (63%) returned the questionnaires, with 728 completed. Questionnaires included the 12-item General Health Questionnaire for assessing depressive symptoms, the Temperament Evaluation of Memphis, Pisa, Paris, and San Diego-Autoquestionnaire version for assessing affective temperaments, the Effort-Reward Imbalance Questionnaire to assess work-related stressors and overcommitment, and questions regarding individual attributes and employment characteristics. Multivariate logistic regression analysis showed that affective temperaments moderated the relationship between work-related stressors and depressive symptoms. Effort (OR = 1.078), which represents job demands and/or obligations imposed on employees, and the upper tertile of overcommitment (OR = 1.589), which represents hyperadaptation to the workplace, were risk factors for depressive symptoms. Additionally, the results for cyclothymic (OR = 11.404) and anxious temperaments (OR = 1.589) suggested that depressive symptoms among this age group may be related to immature-type depression.

## 1. Introduction 

In Japan, the number of domestic patients who had mood disorders with diagnosis codes from F30 (manic episode) to F39 (unspecified mood disorder) in the* International Statistical Classification of Diseases and Related Health Problems 10th Revision *(ICD-10) [1] was 1,041,000 in 2008 [2]. This was 2.4 times higher than in 1996. Employers must pay particular attention to depression in employees because it has a negative impact on productivity [3, 4]. Major depression is a multifactorial disorder [5], and examining risk factors can help employers and managers take measures toward the prevention of depression among workers. 

Previous studies revealed that particular temperaments and personality profiles are risk factors for affective disorders [6]. Other risk factors include sociodemographic characteristics (sex [7, 8], age [7, 9], level of education [8], and marital status [7]), genetic factors [5], stressful life events [5, 10], and previous history of major depression [5]. 

Typus melancholicus, a personality type originally hypothesized by Tellenbach [11], is widely accepted by psychiatrists as a premorbid personality of patients with unipolar depression in Japan and Germany [12–14]. Typus melancholicus is essentially characterized by obsessionality directed to the pursuit of identification with social norms and excessive consideration for people around the individual [11]. Previous studies have revealed that this type of personality may be specifically related to unipolar depression [15–17]. 

Additionally, typus melancholicus may be associated with Japanese type A behavior [18], which is known as a risk factor for coronary heart disease and defined as a set of “behavioral dispositions” such as ambitiousness, aggressiveness, competitiveness, and impatience [19]. In Japan, however, a new type of depression, *immature-type depression*, which is frequently classified as belonging to the bipolar spectrum as defined by Akiskal et al. [20] has increased since the 1990s [21–23]. 

Immature-type depression is commonly observed among adults in their twenties to forties, and the clinical picture involves dependency and aggression towards others (e.g., authority figures) derived from anxiety/agitation and irritation based on patients' immature personality [21, 22]. Abe [23, 24] revealed that patients with immature-type depression do not show typus melancholicus but do exhibit a cyclothymic temperament as defined by Akiskal et al. [20] and experience work-related stressors that cause a sense of frustration that might trigger their immature-type depression [23, 24]. 

According to K. K. Akiskal and H. S. Akiskal [25], affective temperaments play a fundamental role in the predisposition towards affective disorders and affective psychoses, and temperamental dysregulation leads to stressors that can in turn precipitate the development of affective disorders [25]. Akiskal et al. [26, 27] developed the self-rated Temperament Evaluation of Memphis, Pisa, Paris, and San Diego-Autoquestionnaire version (TEMPS-A), a self-assessment tool suitable for measuring affective temperaments. The TEMPS-A, which has increasingly been used to assess affective temperaments, is rooted in an evolutionary biological perspective [28] and has been validated by genetic studies among healthy populations [29–31]. 

Previous epidemiological studies have demonstrated that work-related stressors (e.g., low social support at work, low decision authority, high job demands, and effort-reward imbalance) are risk factors for future psychiatric disorders (e.g., depression) among workers [32–34]. Abe [23] noted that patients with typus melancholicus tend to show a curative effect of pharmacological treatment. However, patients with immature-type depression can relapse even during pharmacological treatment if they return to work without a protective work environment (e.g., positive human relationships) [23].

It is important to focus on workers in their twenties to forties in order to understand affective temperaments as a risk factor for depressive symptoms and immature-type depression. A few studies have examined the influence of temperament and work-related stressors on depressive symptoms in Japanese individuals [35, 36]. However, none have explored whether affective temperaments moderate the relationship between work-related stressors and depressive symptoms among workers. Therefore, the aim of this study is to examine whether affective temperaments assessed by the TEMPS-A moderate the relationship between work-related stressors and depressive symptoms among workers in their twenties to forties.

## 2. Materials and Methods

### 2.1. Participants

Potential participants were all regular employees at six branches of an IT service company providing computer technical support services in Japan. All employees were working in clerical, managerial, or computer technical support positions (*N* = 1382), and most of them were early middle-aged workers who were in their twenties to forties. The male : female ratio was 6 : 4, and the average age was 32.0 years. 

### 2.2. Ethical Considerations and Data Collection

Approval for this study was obtained from the Institutional Ethics Committee at the University of Tokyo (no. 688). Participants were informed in writing on the first page of the questionnaire about the nature of participation (e.g., “participation was voluntary,” and “there was no compensation for participation”) and were assured of confidentiality in the handling of data. Participants were not required to sign consent forms; returning the questionnaire implied consent. 

Self-administered questionnaires were distributed to all employees (*N* = 1382) through the company postal system in July 2004. Of these, 874 employees returned the enclosed questionnaires to the researchers. The response rate of this study (63%) was sufficient compared to response rates of 65% [37] and 49%–68% [35] in previous studies.

The data of 728 complete questionnaires from 297 (41%) males and 412 (59%) females were analyzed. Average ages were 32.74 (SD = 5.99) years and 31.35 (SD = 5.03) years for males and females, respectively. According to statistical data on all workers in the information and communication industry in 2011, published by the secretariat of the Ministry of Economy, Trade and Industry in Japan, the male : female ratio was 7 : 3, and the largest age group was from 25 to 34 years for both sexes [38]. Although the male participation rate was relatively low in this study, the age group distribution was representative of this industry in Japan.

### 2.3. Measures

#### 2.3.1. Depressive Symptoms

To assess depressive symptoms, the Japanese version of the 12-item General Health Questionnaire (GHQ-12) [39] was used, which screens for symptoms of both minor and severe mental disorders [40, 41]. The GHQ-12 has demonstrated high validity, internal consistency, and reliability as well as high sensitivity and specificity in Japanese workers [42]. The participants were asked whether they had recently experienced particular symptoms or behavior related to depression or anxiety. In this study, the GHQ-12 items were scored using the GHQ method because the original scoring is the most valid in its ability to identify psychiatric cases [43]. Each item response category was coded 0-0-1-1. If the answer to an item regarding the presence of a specific depressive type of feeling/symptom represented disagreement (“totally disagree” and “disagree”), it was coded as 0. If the answer indicated agreement (“totally agree” and “agree”), it was coded as 1. The total GHQ-12 score ranges from 0 to 12 points. To identify clinical problems associated with depressive symptoms, binary data were used calculated by a cutoff point of 3/4, where 0 indicated a total score of 3 points or less (meaning low risk of depressive symptoms), and 1 indicated a total score of 4 points or more (meaning high risk of depressive symptoms) [44, 45]. 

#### 2.3.2. Affective Temperaments

Affective temperaments were assessed by the self-rated Temperament Evaluation of Memphis, Pisa, Paris, and San Diego-Autoquestionnaire version (TEMPS-A) developed by Akiskal et al. [26, 27]. The TEMPS-A comprises 110 items measuring affective temperaments that define the bipolar spectrum, with depressive, cyclothymic, hyperthymic, irritable, and anxious subscales [26, 27]. 

The TEMPS-A subscales measure not only emotional, cognitive, psychomotor, and circadian traits that might predispose one to major mood disorders, but also positive characteristics that could play an adaptive role in an evolutionary context [26] (see Table 6). 

The TEMPS-A has been translated into 25 languages and validated across many cultures, including among Japanese adults [37, 46, 47], and concurrent validity against the Temperament and Character Inventory has been shown [26]. Participants were asked if they had particular ideas or attitudes regarding temperament. For each answer, a “no” was scored as 1 and a “yes” as 2. These scores were added and divided by the number of related questions to represent each temperament score. 

#### 2.3.3. Work-Related Stressors and Overcommitment

To assess work-related stressors and overcommitment, the Japanese short version of the Effort-Reward Imbalance (ERI) Questionnaire developed by Siegrist was included [48, 49]. High internal consistency, discriminant validity, and factorial structure of the ERI questionnaire scale were confirmed for a range of working populations in five European countries (*N* = 18,943) [49]. The Japanese short version of the questionnaire, which has been culturally adapted and translated, has high internal reliability and validity for the Japanese working population [50–52]. 

The ERI questionnaire consists of two scales for measuring extrinsic components and one scale for measuring an intrinsic overcommitment component. The extrinsic components measured are *effort* (6 Likert-scale items), which refers to the demanding conditions of the work environment that employees face; *reward*, (11 Likert-scale items with three subscales), which refers to the three transmitters considered to be sources of reward for employees (money, esteem, and career opportunities). The overcommitment scale (6 Likert-scale items), which is an intrinsic personal dimension of ways of coping with demanding situations and of eliciting extrinsic rewards, is the intrinsic overcommitment component in the ERI questionnaire [48, 50]. 

Items measuring the extrinsic components were quantified in two steps using Siegrist's ERI scale and scoring method. First, participants agreed or disagreed with an item describing a typical work-related situation or experience. For effort items (e.g., “I have constant time pressure due to a heavy workload”), “no” (participants disagreed with the work-related situation or experience) answers were scored as 1. Participants who answered “yes” (participants agreed) were also asked to rate their degree of experienced distress on a scale from 2 (I am not at all distressed) to 5 (I am very distressed). Potential scores for effort ranged from 6 to 24, with higher total scores indicating greater participant effort.

For items related to reward (e.g., “My job promotion prospects are poor”), “no” (participants disagreed that their situation involved a lack of reward) answers were scored as 5. Participants who answered “yes” (participants agreed) were also asked to rate their degree of distress on a scale from 4 (I am not at all distressed) to 1 (I am very distressed). Potential scores for reward ranged from 11 to 44. The lower the total score, was the more the participant was feeling stressed about rewards. 

To assess the intrinsic overcommitment component, participants were asked to state their degree of agreement with six declarative statements (e.g., “I usually take criticism very seriously”) on a 4-point scale ranging from 1 (strongly disagree) to 4 (strongly agree). The theoretical range for the overcommitment scale is 4–24, with higher values indicating that participants are easily overwhelmed by time pressure at work or have problems relaxing and disconnecting from work during time off. The total score with upper tertiles was dichotomized in accordance with the Japanese version of the ERI questionnaire [52]. In the analysis, binary data were used: a value of 1 indicated the presence of overcommitment, whereas a value of 0 indicated a favorable condition (no overcommitment) in the participants.

Additionally, data on individual attributes (sex, age, marital status, and educational level) and employment characteristics (type of job, shift work, mean working hours per day, and frequency of working on days off per month) were collected. 

### 2.4. Data Analyses

Descriptive statistics and Cronbach's alpha coefficients for independent variables (e.g., ERI, overcommitment, and TEMPS-A) and the GHQ-12, the dependent variable, were calculated. As a preliminary analysis, the coefficient of association between each independent variable and the dependent variable was calculated (Phi coefficient or Cramer's measure of association) to compare high-risk and low-risk groups of depressive symptoms. Additionally, Spearman's correlation coefficients between the GHQ-12 and independent variables were calculated. Finally, to provide tangible data relating to factors associated with depressive symptoms, a multivariate logistic regression analysis for the GHQ-12 was performed. 

In model 1, the subscales of the Japanese version of the ERI questionnaire (effort, reward, and overcommitment) were added to the equation as well as the independent variables that showed a significant relationship with the GHQ-12 in the preliminary analysis. Considering an interaction effect over the sum of the separate effects of effort and reward, data for the interaction of effort and reward were added to the equation. To examine the effect of temperament on the GHQ-12, in model 2, the five temperament variables (from the TEMPS-A) were entered into the equation. In both models, the odds ratios (ORs) and 95% confidence intervals (CIs) for the independent variables that were significantly associated with the GHQ-12 in the preliminary analysis were also calculated. The statistics package used was SPSS 15.0J (SPSS Japan Inc., Tokyo, Japan). 

## 3. Results

Among the participants, 327 (45%) had a university or higher degree. One hundred twenty-four participants (17%) were working more than 10 hours per day, and 147 (20%) were working on days off more than one day per month, on average (see Table 1).

Table 2 presents descriptive statistics and Cronbach's alpha coefficients for the dependent and independent variables. Cronbach's alpha coefficients for effort, reward, and overcommitment were 0.80–0.9. Coefficients for the five temperaments ranged from 0.69 to 0.89.

Table 3 presents the coefficients of association between each independent variable and the GHQ-12 (e.g., high-risk group and low-risk group). There were significant associations between the GHQ-12 and average working hours per day and frequency of working on days off per month. 

Table 4 presents Spearman's correlation coefficients for each independent variable and the total GHQ-12 scores. All independent variables were significantly associated with the dependent variable. The highest correlation with the GHQ-12 was the cyclothymic temperament (*r* = 0.293), and the second highest was the anxious temperament (*r* = 0.282). 

Table 5 presents the results of the multiple logistic regression analysis for the GHQ-12. In model 1, a high risk of depressive symptoms (GHQ-12 score of 4 points or more) was significantly associated with the following variables: female sex (OR = 1.461, 95% CI 1.022–2.089), age (OR = 0.964, 95% CI 0.936–0.994), effort (OR = 1.085, 95% CI 1.032–1.140), and upper tertile of overcommitment (OR = 2.113, 95% CI 1.409–3.169). In model 2, a high risk of depressive symptoms was significantly associated with the following variables: effort (OR = 1.078, 95% CI 1.023–1.135), upper tertile of overcommitment (OR = 1.589, 95% CI 1.015–2.485), the cyclothymic temperament (OR = 11.404, 95% CI 2.996–43.409), and the anxious temperament (OR = 1.589, 95% CI 1.015–2.485).

## 4. Discussion

### 4.1. Affective Temperaments as a Predictor of Depressive Symptoms among Workers in Their Twenties to Forties

Findings revealed that affective temperaments moderated the relationship between work-related stressors and depressive symptoms among workers in their twenties to forties. Additionally, the cyclothymic and anxious temperaments were high-risk factors for depressive symptoms after adjustment for sociodemographic variables and employment characteristics. This is a remarkable result, clarifying that temperament exerts a more important influence on depressive symptoms of workers in their twenties to forties than work-related predictors such as effort and the upper tertile of overcommitment.

A previous study examining the influence of affective temperaments assessed by the TEMPS-A and work-related stressors on employee depression in the information and communication industry revealed that predictors of depression were different between male and female workers [36]. However, sex was not a risk factor in the present findings. Among independent variables, the correlation between GHQ-12 and the cyclothymic temperament had the highest coefficient followed by the anxious temperament. This is in agreement with the findings of a previous study of undergraduate students aged 18–30 [53]. Cyclothymic and anxious temperaments may be common premorbid risk factors for depression among healthy young people (e.g., undergraduate students and workers in their twenties to forties). 

Additionally, this study's findings suggest that participants at high risk for depressive symptoms may have been those with immature-type depression, in which cyclothymia is the premorbid personality [23]. Japanese researchers have found that patients with immature-type depression are more commonly confirmed among workers in their twenties to forties, are in the bipolar II category [23], and tend to show the cyclothymic temperament [23, 54, 55]. Cyclothymia is the core feature of the bipolar mood disorder spectrum [20]. In previous studies, the cyclothymic temperament assessed by the TEMPS-A showed a significant association with the s allele of 5-HTTLPR [29, 30], which has been associated with affective disorders [56] and subthreshold forms of depression [57]. 

Furthermore, a previous study reported that the anxious temperament assessed by the TEMPS-A is highly correlated (*r* = 0.759) with neuroticism assessed by the Neuroticism, Extraversion, Openness, Five-Factor Inventory (NEO-FFI) [58]. The present finding that the anxious temperament was a high-risk factor for depressive symptoms supports that of a previous study of white-collar workers in Japan [35]. That study examined predictors of first onset of major depressive episodes, including work-related stress and temperaments assessed by the NEO-FFI. It revealed that neuroticism and overprotection were associated with the primary onset of depression among workers [35]. 

Psychosocial factors can protract immature-type depression. Thus, an understanding of the sociocultural background of patients with this type of depression is important from the viewpoint of treatment [21]. Abe noted that in modern Japanese society following the postwar period of high economic growth, children have been raised with a lack of paternal authority and conformity to social norms compared to those raised a few generations ago in traditional communities in Japan. As a result, children tend to be narcissistic and dependent with a strong maternal attachment [21].

Patients with immature-type depression tend to overestimate their working ability and are not norm oriented. The clinical picture indicates dependency and aggression towards others derived from anxiety/agitation and irritation based on their immature personality. Thus, these patients can become depressed following trivial events at the workplace [21–24]. They can recover from depression if they reenter protective work environments, but if they do not, repeated depressive phases will likely occur [23]. 

To prevent immature-type depression in high-risk individuals showing the cyclothymic temperament, it is important to understand their immaturity and help them become more independent in relationships, thus improving work adjustment [21, 23]. As a basic remedy for immature-type depression, detecting the affective temperaments of workers who are at high risk for depressive symptoms through screening and providing supportive psychotherapy from mental health professionals related to work adjustment can be effective in conjunction with pharmaceutical therapy [23]. 

### 4.2. Work-Related Predictors of Depressive Symptoms among Workers in Their Twenties to Forties

In this study, although its odds ratio was lower than for affective temperaments, effort predicted depressive symptoms among workers in their twenties to forties after adjusting for sociodemographic and employment characteristics. In agreement with this finding, two previous studies revealed that high effort, representing job demands and/or obligations imposed on employees [48, 59], was a risk factor for depression in workers assessed by the GHQ [33, 60]. Additionally, psychological job demand was a significant predictor of subsequent depressive symptoms [61, 62]. Together, these study findings suggest that the assessment of quantitative and qualitative workload to prevent the occurrence of depression among workers in their twenties to forties is important, regardless of the workers' temperaments.

In this study, the upper tertile of overcommitment was an independent, high-risk factor for depressive symptoms among participants, consistent with a previous review of 45 studies [59]. That review study revealed that employees characterized by high overcommitment were 1.92–5.92 times more likely to suffer from various psychosomatic symptoms (e.g., depression) than less overcommitted employees. In addition, findings showed that overcommitment can have a direct effect on adverse health outcomes of employees [59]. 

Overcommitment results from excessive effort and perceptual distortion (in particular, an underestimation of challenges and overestimation of coping resources) are based on type A behavior [18, 59] that may be triggered by an underlying motivation to experience recurrent esteem and approval [48, 63]. Fukunishi et al. (1992) posited that typus melancholicus may be involved in Japanese type A behavior [18], defined as a set of “behavioral dispositions such as ambitiousness, aggressiveness, competitiveness, impatience, and emotional responses (e.g., irritability) [19]. 

Typus melancholicus embodies the very high demands of one's work ethic (e.g., concern with orderliness, conscientiousness, exaggerated sense of order, and elevated demand above one's average abilities) [11, 64]. Additionally, Sakai et al. [37] revealed that typus melancholicus is significantly correlated with job stress (e.g., change in work, quantitative workload, and role ambiguity) as well as affective temperaments assessed by the TEMPS-A, and that it manifests in workers as hyperadaptation to the workplace. 

Although this study did not examine its effect, typus melancholicus might have influenced the results regarding overcommitment, via the mechanism of hyperadaptation to the workplace. Further studies are needed to examine the influence of typus melancholicus, affective temperaments assessed by the TEMPS-A, and overcommitment on depressive symptoms among workers in their twenties to forties. Given the current findings, it is suggested that preventive interventions (e.g., cognitive-behavioral therapy) may assist workers in adopting more constructive coping patterns when exposed to stressful work circumstances as well as help them reflect on the ideas and assumptions that drive overcommitment [63]. 

### 4.3. Study Limitations and Directions for Future Research

This study has some limitations. First, problems unique to workers in a company in the information and communication industry, of whom 87% were computer technical support staff, may be reflected in the results. Additionally, the findings might have been different in a clinical Japanese sample or a cohort of healthy employees from a different cultural background. Second, the present findings are based on data from 2004 and thus should be interpreted with caution. However, this study reveals new insights into Japanese workers in the age group most vulnerable to immature-type depression that other studies have not provided. Third, different methods of measuring temperament, personality traits, and work-related stressors may have influenced the results, perhaps causing the inconsistencies between this study and those conducted previously [35, 36]. In the future, it would be beneficial to conduct a cohort study in other occupational groups in different countries that examines affective temperaments measured by the TEMPS-A associated with work-related stressors and depressive symptoms among workers in their twenties to forties.

## 5. Conclusions 

This research explored whether affective temperaments moderate the relationship between work-related stressors and depressive symptoms. It targeted workers in their twenties to forties, a group in which immature-type depression is commonly observed in Japan. Findings revealed that affective temperaments (cyclothymic and anxious) were high-risk factors for depressive symptoms. Additionally, high effort, which represents job demands and/or obligations imposed on the employees, and overcommitment or hyperadaptation to the workplace were also risk factors for depressive symptoms. These findings suggest that depressive symptoms among this age group may be indicative of immature-type depression, in which cyclothymia is the premorbid personality and classified in the bipolar II spectrum. For the prevention of immature-type depression in high-risk individuals showing the cyclothymic temperament, it is important to understand their immaturity and help them become more independent, especially in regard to human relations, thus contributing to better work adjustment.

## Figures and Tables

**Table 1:** Sociodemographic characteristics of the sample (*N* = 728).

Variables	*N*	%
(1) Sex		
Male	297	41
Female	431	59
(2) Mean age ± SD		
Male	32.74 (±5.99)	
Female	31.35 (±5.03)	
(3) Marital status		
Single	222	30
Married	506	70
(4) Education		
Junior college or vocational school equivalency degree	401	55
College graduate or higher	327	45
(5) Type of job		
Clerical post	81	11
Computer technical support	636	87
Managerial post	11	2
(6) Shift work		
Without	252	35
With	476	65
(7) Mean working hours per day		
Less than seven or fewer hours per day	59	8
Eight hours per day	384	53
Nine hours per day	161	22
Ten hours per day	94	13
More than eleven hours per day	30	4
(8) Frequency of working on days off		
Non	581	80
More than one day on a monthly basis	147	20

**Table 2:** Descriptive statistics and Cronbach's alpha coefficients of dependent and independen variables (*N* = 728).

Variables	Mean	SD	Items	Range	The Cronbach's alpha
GHQ-12^(1)^	29.77	5.56	12	17–48	0.84
GHQ-12^(2)^	4.66	2.20	12	0–11	0.48
Effort^(3)^	13.69	4.74	6	6–30	0.87
Rewards^(3)^	39.92	8.29	11	11–55	0.91
Overcommitment^(3)^	13.60	3.48	6	6–24	0.80
Depressive^(4)^	1.42	0.17	21	1.00–1.86	0.69
Cyclothymic^(4)^	1.30	0.22	21	1.00–2.00	0.85
Hyperthymic^(4)^	1.27	0.18	21	1.00–1.86	0.78
Irritable^(4)^	1.18	0.18	21	1.00–1.86	0.85
Anxious^(4)^	1.24	0.21	26	1.00–1.96	0.89

^(1)^Scored using the Likert method (1-2-3-4).

^(2)^Scored using the GHQ method (0-0-1-1).

^(3)^Subscales of the Japanese short version of Effort-Reward Imbalance scale.

^(4)^Affective temperaments assessed by the TEMPS-A.

**Table 3:** Results of coefficient of association between each control variables and GHQ-12 (*N* = 728).

Variables	GHQ-12^(1)^			
	Low risk	High risk	Coefficient of association^(2)^	*P* value
	(*n* = 93)	(*n* = 537)		
(1) Sex				
Female	81	216	−0.051	0.170
Male	138	293		
(2) Marital status				
Single	75	147	0.053	0.149
Married	144	362		
(3) Education				
Junior college or vocational school equivalency degree	121	280	0.002	0.952
University graduate or higher	98	229		
(4) Type of job				
Clerical post	27	54	0.061^(3)^	0.258
Computer technical support	191	445		
Managerial post	1	10		
(5) Shift work				
Shift work with night shifts	79	173	0.020	0.588
Without night shifts	140	336		
(6) Average working hours per day				
Nine hours or less	190	414	0.066	0.074
Ten hours or more	29	95		
(7) Frequency of working on days off per month				
None	188	393	7.084	0.008
More than one day per month	31	116		

^(1)^High risk is those who scored 4 or more when the cut-off point of the GHQ-12 was 3/4. Low risk is those who scored 3 or less.

^(2)^Phi coefficient.

^(3)^Cramer's measure of association.

**Table 4:** Correlation coefficients of independent and dependent variables (n=728)^(1)^.

	(1)	(2)	(3)	(4)	(5)	(6)	(7)	(8)	(9)	(10)
(1) GHQ-12^(2)^	1									
(2) Effort	0.239***									
(3) Rewards	−0.180***	−0.518***								
(4) Overcommitment	0.272***	0.467***	−0.384***							
(5) Upper tertile of overcommitment^(3)^	0.242***	0.428***	−0.341***	0.846***						
(6) *Depressive * ^(4)^	0.202***	0.233***	−0.276***	0.438***	0.408***					
(7) *Cyclothymic * ^(4)^	0.293***	0.210***	−0.169***	0.328***	0.295***	0.501***				
(8) *Hyperthymic * ^(4)^	0.101**	0.049 n.s.	−0.007 n.s.	−0.061 n.s.	−0.032 n.s.	−0.111**	0.226***			
(9) *Irritable * ^(4)^	0.204***	0.225***	−0.213***	0.334***	0.305 ***	0.421***	0.686***	0.206***		
(10) *Anxious * ^(4)^	0.282***	0.287***	−0.246***	0.531***	0.459 ***	0.582***	0.622***	0.020 n.s.	0.640***	1

^(1)^Spearman correlation coefficient. Symbols indicate level of significance: *P < 0.05; **P < 0.01; ***P < 0.001.

^(2)^1 = when the cut-off point of the GHQ-12 was 3/4, those who scored 4 or more; 0 = those who scored 3 or less.

^(3)^1 = those who are scored in the upper 30th percentile; 0 = others.

^(4)^Affective temperaments assessed by the TEMPS-A.

**Table 5:** Multivariate logistic regression model for the GHQ-12 (*N* = 728).

Variables	Model 1^(1,2)^			Model 2^(1,3)^		
	OR	95% CI	*P* value	OR	95% CI	*P* value
Sex (reference category = female)	1.461	1.022–2.089	0.037	1.366	0.943–1.979	0.099
Age	0.964	0.936–0.994	0.019	0.977	0.947–1.009	0.157
Frequency of working on days off per month (reference category = one day or more)	1.551	0.965–2.492	0.070	1.372	0.837–2.249	0.210
Average working hours per day (reference category = 10 hours or more)	0.796	0.466–1.359	0.403	0.795	0.456–1.385	0.418
Effort	1.085	1.032–1.140	0.001	1.078	1.023–1.135	0.005
Rewards	0.975	0.949–1.001	0.056	0.978	0.951–1.006	0.116
Effort*Rewards	1.004	0.999–1.009	0.087	1.004	0.999–1.009	0.140
Upper tertile of overcommitment (reference category = present)	2.113	1.409–3.169	<0.001	1.589	1.015–2.485	0.043
*Depressive* ^ (4)^				0.624	0.164–2.368	0.488
*Cyclothymic* ^ (4)^				11.404	2.996–43.409	<0.001
*Hyperthymic* ^ (4)^				2.605	0.887–7.647	0.081
*Irritable* ^ (4)^				0.337	0.067–1.701	0.188
*Anxious* ^ (4)^				6.409	1.484–27.684	0.013

^(1)^OR: odds ratio; Cl: confidence interval.

^(2)^Model 1: Hosmer-Lemeshow goodness of fit *χ* ^2^ = 3.484, df = 8, *P* = 0.900.

^(3)^Model 2: Hosmer-Lemeshow goodness of fit *χ* ^2^ = 6.072, df = 8, *P* = 0.639.

^(4)^Affective temperaments assessed by the TEMPS-A.

**Table 6:** Characteristics of each temperament.

Temperament	Characteristics of each temperament
Depressive temperament	Skeptical, hypercritical or complaining, conscientious or self-disciplining, self-critical, self-reproaching or self-derogatory, gloomy, pessimistic, humourless or incapable of fun, preoccupied with inadequacy, failure and negative events to the point of morbid enjoyment of one's failures, and brooding and given to worry.

Cyclothymic temperament	Introverted self-absorption alternating with uninhibited people seeking, marked unevenness in quantity and quality of productivity-associated unusual working hours, shaky self-esteem alternating low self-confidence and overconfidence, biphasic dysregulation characterized by abrupt endoreactive shifts from one phase to the other, each phase lasting for few days at a time, with infrequent euthymia, unexplained tearfulness alternating with excessive punning and jocularity, decreased verbal output alternating with talkativeness, mental confusion alternating with sharpened and creative thinking, and hypersomnia alternating with decreased need for sleep.

Hyperthymic temperament	Cheerful, overoptimistic or exuberant, vigorous, full of plans, improvident, carried away by restless impulses, overtalkative, warm, people-seeking or extroverted, uninhibited, stimulus-seeking or promiscuous, naïve, overconfident, self-assured, boastful, bombastic or grandiose, overinvolved, and meddlesome.

Irritable temperament	Habitually moody irritable and choleric with infrequent euthymia, impulsive, obtrusiveness, tendency to brood, dysphoric restlessness, indeterminate early onset, and ill-humored joking.

Anxious temperament	Worry, vigilance, tension, oversensitive, unrestful sleep, and gastrointestinal symptoms.

The characteristics of each TEMPS-A temperament adapted from previous studies [25–27].
